# Patterns of recurrence after low-dose postoperative radiotherapy for head and neck squamous cell carcinoma

**DOI:** 10.1186/s43046-021-00098-w

**Published:** 2021-12-20

**Authors:** Wataru Makino, Joichi Heianna, Kazuki Ishikawa, Takeaki Kusada, Hitoshi Maemoto, Takuro Ariga, Akira Matayoshi, Toshiyuki Nakasone, Hitoshi Hirakawa, Shinya Agena, Yukashi Yamashita, Hiroyuki Maeda, Sadayuki Murayama

**Affiliations:** 1 Department of Radiology, Graduate School of Medical Science, University of the Ryukyus, 207 Uehara, Nishihara, Okinawa, 903-0215 Japan; 2 Department of Oral and Maxillofacial Surgery, University Hospital of the Ryukyus, Nishihara, Okinawa, 903-0215 Japan; 3 Department of Otorhinolaryngology, Head and Neck Surgery, Graduate School of Medicine, University of the Ryukyus, 207 Uehara, Nishihara, Okinawa, 903-0215 Japan

**Keywords:** Head and neck squamous cell carcinoma, Postoperative radiation therapy, Patterns of recurrence

## Abstract

**Background:**

Postoperative chemoradiotherapy is recommended for patients with head and neck squamous cell carcinoma with positive margins or extracapsular extension at high risk of recurrence. However, high-dose radiotherapy in the head and neck region often causes severe acute and late radiation-related adversities. In our institution, the radiation dose has been relatively lower than that used in Western countries to reduce radiation-related toxicities. Therefore, in this study, we examined the treatment outcomes of low-dose postoperative chemoradiotherapy.

**Methods:**

The outcomes of 90 consecutive head and neck squamous cell carcinoma patients who received postoperative radiotherapy between June 2009 and December 2016 were retrospectively analyzed. All patients received postoperative three-dimensional conformal radiotherapy with or without concurrent systemic chemotherapy. The median patient age was 65 years. Concurrent chemoradiotherapy was administered at a total dose of 50.4 Gy in 28 fractions (daily fraction, 1.8 Gy). High-risk patients received 10.8 Gy of boost irradiation in six fractions. For radiotherapy alone, the irradiation dose was up to 54 Gy in 30 fractions and 64.8 Gy in 36 fractions for high-risk patients to increase the treatment intensity.

**Results:**

The median follow-up period was 40.5 months. The 3-year locoregional control and overall survival rates were 67.5% and 82.7%, respectively. A significantly higher proportion of patients with oral cavity carcinoma experienced locoregional failure (*p* = 0.004). The acute adverse events were mild, and the only late adverse event was grade 3 dysphagia (*n* = 3).

**Conclusion:**

This study suggests that de-escalation of the postoperative radiation dose can potentially reduce the severe adverse events of irradiation in patients while ensuring its effectiveness. In patients with oral cavity carcinoma, it might be necessary to increase the radiation dose.

## Background

Two essential trials tested the benefit of postoperative chemoradiotherapy in patients with head and neck squamous cell carcinoma (HNSCC). In 2004, the European Organization for Research and Treatment of Cancer (EORTC) and Radiation Therapy Oncology Group (RTOG) published the results of two phase III trials (EORTC 22931 and RTOG 95–01). The studies compared concurrent postoperative chemoradiotherapy using tri-weekly 100 mg/m^2^ cisplatin (CDDP) with postoperative radiotherapy alone [1, 2]. Radiotherapy administered in both arms consisted of 60 Gy with or without a 6-Gy boost (RTOG) or administration of 66 Gy (EORTC) [3]. Although the definitions of risk factors for recurrence in the two trials differed, Bernier et al. conducted a comparative analysis using data pooled from the EORTC 22931 and RTOG 95–01 studies to identify which patients require adjuvant concomitant chemoradiotherapy following surgery. They concluded that close and/or positive margins at the site of resection of the primary lesion and extracapsular extension (ECE) of nodal metastasis were the most significant predictors of poor outcomes [3]. Therefore, postoperative chemoradiotherapy with CDDP is recommended for patients with HNSCC with positive margins or ECE at high risk of recurrence. The current National Comprehensive Cancer Network (NCCN) guidelines recommend a radiation dose of 60–66 Gy (2 Gy/fraction) for high-risk areas and 44–50 Gy (2 Gy/fraction) to 54–63 Gy (1.6–1.8 Gy/fraction) for low-to-intermediate-risk areas [4]. However, high-dose radiotherapy in the head and neck region often causes severe dermatitis and mucositis during treatment. This treatment can also cause late adverse events such as xerostomia, loss of the sense of taste, and long-term percutaneous endoscopic gastrostomy (PEG) tube dependence, which are highly distressing. In our institution, the radiation dose for patients with postoperative HNSCC has been relatively lower than that used in Western countries in an attempt to reduce acute and late radiation-related toxicities. Therefore, in this study we examined the treatment outcomes of low-dose postoperative chemoradiotherapy.

## Methods

### Study design and institutional review board approval

We performed a retrospective chart review of patients with HNSCC who received postoperative radiotherapy with or without chemotherapy. This single-center, retrospective study was approved by our institutional ethical review board (Permission number: 1639).

### Patients

**Fig. 1:**
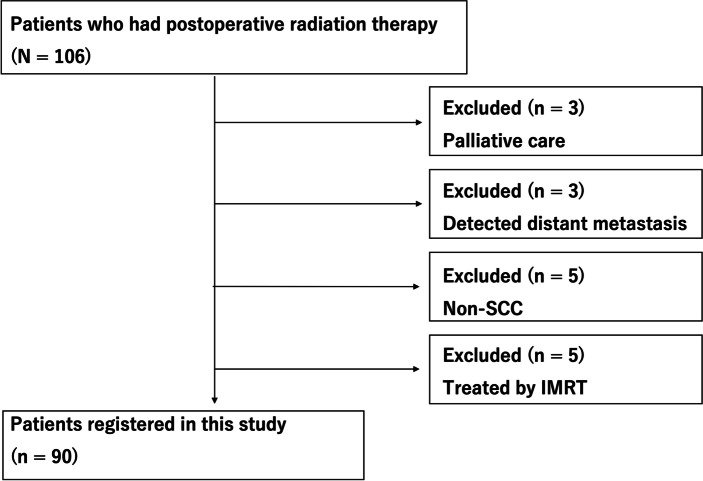
Flowchart of the study sample. SCC, squamous cell carcinoma; IMRT, intensity-modulated radiation therapy

In this study, we included patients with head and neck cancer who underwent radical surgery followed by radiotherapy at our hospital between June 2009 and December 2016, regardless of any risks. Patients whose general conditions deteriorated before radiotherapy or patients who had distant metastases detected before radiotherapy were excluded. Furthermore, we excluded patients with adenoid cystic carcinoma or sarcoma considering the fact that we targeted squamous cell carcinoma. We also excluded patients who underwent intensity-modulated radiation therapy (IMRT) because IMRT, which had been recently introduced in our institution during the study period, was not widely used, especially in the postoperative period. Assessing the impact of IMRT in the small number of patients who received IMRT would have been difficult; therefore, only patients who received three-dimensional conformal radiotherapy (3D-CRT) were included in the current study. A total of 106 consecutive patients were registered, and eventually, 90 patients were enrolled in the study (Fig. 1). All patients underwent computed tomography (CT), magnetic resonance imaging, and endoscopy of the head and neck as pretreatment evaluations before undergoing surgery. Positron emission tomography was performed if necessary.

### Radiotherapy

All radiotherapy plans were conducted using a volumetric CT-based three-dimensional treatment planning system (Eclipse™; Varian Medical System, Inc.). The patients were treated in the supine position using a thermoplastic mask for immobilization. Radiotherapy was prescribed in 1.8-Gy fractions with 4- or 6-MV photons from a linear accelerator (Clinac® iX; Varian Medical Systems, Inc.) in three-dimensional conformal radiotherapy (3DCRT). For each patient, the radiation field and dose were formulated with the input of a multidisciplinary team that included a radiation oncologist, head and neck surgeon, and radiologist. Target volumes were defined as follows: the high-risk clinical target volume (CTV) was defined as areas considered at high risk of recurrence for a positive surgical margin and/or lymph node with ECE, based on pathological findings. The prophylactic CTV was defined as areas that included the entire operative bed and elective lymph node levels, including the levels II–V nodes, supraclavicular nodes, and retropharyngeal nodes. In the patients with oral cavity cancers, levels Ia and Ib were also included electively. Circumferential 5-mm expansion of the corresponding CTV gave rise to the planning target volume. For concurrent chemoradiotherapy, a total dose of 50.4 Gy was delivered in 28 fractions to the prophylactic area. High-risk patients received 10.8 Gy of boost irradiation in 6 fractions to the high-risk areas. For radiotherapy alone, the irradiation dose was up to 54 Gy in 30 fractions and 64.8 Gy in 36 fractions for high-risk patients to increase the treatment intensity.

### Chemotherapy

The patients were treated with concurrent systemic therapy in accordance with the recommendation of the multidisciplinary team in consideration of clinicopathologic factors and patient characteristics. Chemotherapy mainly consisted of CDDP or nedaplatin (CDGP). CDDP was administered at a dose of 100 mg/m^2^ every 3 weeks. CDGP is a CDDP analog developed to decrease the toxicities induced by the parent drug, such as nephrotoxicity and gastrointestinal toxicity [5]. CDGP was administered at a dose of 90 mg/m^2^ every 4 weeks. In some cases, we also added 5-fluorouracil 800 mg/m^2^ or docetaxel 60 mg/m^2^. Carboplatin was used for patients with impaired renal function. We performed radiation therapy alone only at patients with specific conditions such as severe renal or hepatic dysfunction or very elderly patients.

### Evaluation

During treatment, adverse events were graded according to the Common Terminology Criteria for Adverse Events version 4.0. After completing the treatment, the patients were followed up every 1–2 months for the first year and every 3–4 months for 4 successive years. The current study endpoints included overall survival (OS), disease-free survival (DFS), locoregional control (LRC), and adverse events.

### Statistical analysis

Time-to-event analyses were performed using the Kaplan–Meier method for several outcome measures. OS was calculated from the initiation of postoperative radiation to death from any cause. DFS was calculated from the initiation of postoperative radiation to any relevant events, including local, regional, and distant recurrence. LRC was calculated from the initiation of postoperative radiation to local and/or regional recurrence. For DFS and LRC, patients who died without experiencing any of these events were censored at the time of the last follow-up. To identify the clinicopathological factors influencing treatment outcome, univariate and multivariate analyses were performed using Cox regression analysis. The statistical significance of differences between the groups was evaluated using the log-rank test. The level of statistical significance was set at *p* < 0.05. All statistical analyses were conducted using EZR ver. 1.41 (Saitama Medical Center, Jichi Medical University, Saitama, Japan), a graphical user interface for R (The R Foundation for Statistical Computing, Vienna, Australia). More precisely, it is a modified version of R commander designed to add statistical functions frequently used in biostatistics [6].

## Results

### Patient characteristics

The outcomes of 90 consecutive patients who received postoperative radiotherapy for HNSCC between June 2009 and December 2016 were analyzed. The patients’ characteristics are summarized in Table 1. Of the patients, 74 (82%) were men and 16 (18%) were women, with a median age of 65 years (range, 27–88 years). The distribution of the primary disease sites was as follows: oral cavity, 50 (56%) patients; hypopharynx, 27 (30%); oropharynx, 9 (10%); and larynx, 4 (4%). Among the nine patients with oropharyngeal carcinoma, only one patient was p16-positive whereas four patients were p16-negative and the status was unknown in the remaining four patients. The clinical stages of the patients were distributed as follows: stage II disease, 1 patient (1%); stage III disease, 10 (11%); stage IVA disease, 78 (87%); stage IVB disease, 1 (1%). Concerning the histopathological findings, 17 patients (19%) had an ECE in the resected lymph nodes, 8 (9%) had a positive margin at the resection site of the primary disease, and 21 (23%) had either a positive margin or an ECE.

**Table 1:** Patient characteristics (*n* = 90)

Age	
Median (range)	65 (27–88)
Sex	
Male	74 (82%)
Female	16 (18%)
Primary site	
Oral cavity	50 (56%)
Hypopharyngeal	27 (30%)
Oropharyngeal	9 (10%)
p16 positive	1
p16 negative	4
p16 status unknown	4
Laryngeal	4 (4%)
Stage	
II	1 (1%)
III	10 (11%)
IVa	78 (87%)
IVb	1 (1%)
T classification	
T1	13 (14%)
T2	20 (22%)
T3	21 (23%)
T4	36 (40%)
N classification	
N0	7 (8%)
N1	12 (13%)
N2a	2 (2%)
N2b	47 (52%)
N2c	21 (23%)
N3	1 (1%)
Extracapsular extension	
Yes	17 (19%)
No	73 (81%)
Positive/close margin	
Yes	8 (9%)
No	82 (91%)

### Radiotherapy

The treatment details are summarized in Table 2. The median interval from the last surgery to the initiation of postoperative radiotherapy was 42 days (range, 14–77 days). All patients were able to complete their scheduled radiation treatment. The median prescribed dose was 50.4 Gy (range, 50.4–66.6 Gy). Eighty-four patients (93%) underwent prophylactic bilateral neck irradiation. Six elderly and fragile patients (7%) received unilateral neck irradiation because both the primary tumor and lymph nodes were confined to one side. Most patients received the dose as per protocol. Nevertheless, 2 patients with hypopharynx and tongue cancers received 66.6 Gy of radiation owing to the presence of a very high risk of recurrence, and the multidisciplinary team recommended a dose increase.

**Table 2:** Treatment details (*n* = 90)

Radiation dose (Gy)		
Median (range)	50.4 (50.4–66.6)	
Radiation field		
Bilateral	65 (72%)	
Bilateral + boost	19 (21%)	
Unilateral	5 (6%)	
Unilateral + boost	1 (1%)	
Boost irradiation		
Yes	20 (22%)	
No	70 (78%)	
Concurrent chemotherapy		
Yes	68 (76%)	
No	22 (24%)	
Chemotherapy regimen		
CDGP + 5FU	32 (47%)	
CDDP	13 (19%)	
CDDP + 5FU	9 (13%)	
CDGP + DTX	6 (9%)	
DTX	5 (7%)	
CBDCA	2 (3%)	
CDDP + DTX	1 (2%)	

*CDGP* nedaplatin, *5FU* 5-fluorouracil, *DTX* docetaxel, *CDDP* cisplatin, *CBDCA* carboplatin

### Chemotherapy

Sixty-eight patients (76%) underwent concurrent chemotherapy with postoperative radiotherapy. The most common chemotherapy regimen was CDGP + 5FU in 32 patients (47%), followed by CDDP alone in 13 patients (19%). The anti-cancer agents used in this study were diverse because the otolaryngology or oral surgery departments handled chemotherapy.

### Treatment outcomes

#### OS, DFS, and LRC

**Fig. 2:**
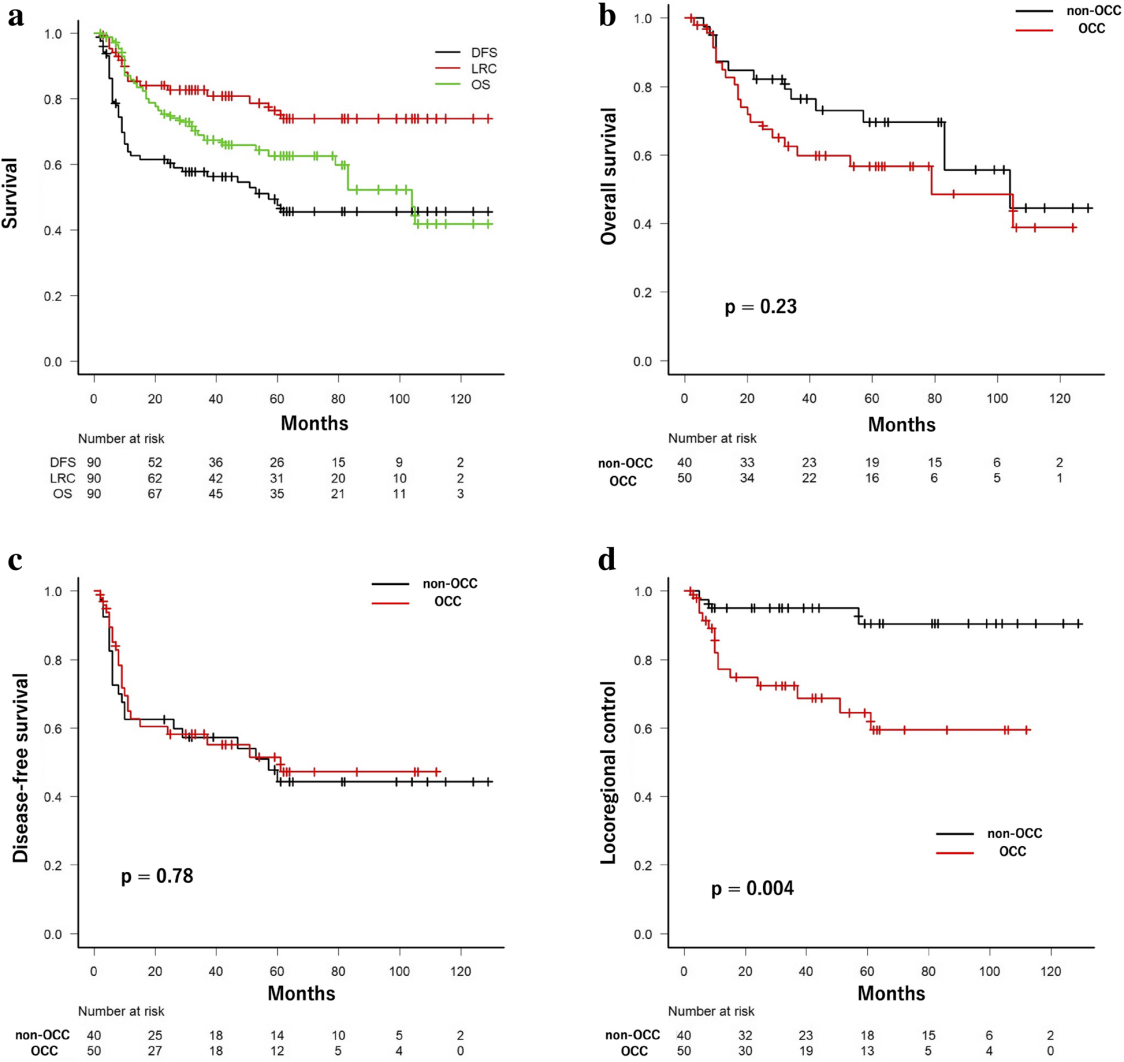
Treatment outcomes estimated using the Kaplan–Meier method. **A** Kaplan-Meier curves of overall survival (OS), disease-free survival (DFS), and locoregional control (LRC). **B** OS stratified by the group of patients with OCC (oral cavity carcinoma) or those without. **C** DFS stratified by the group of patients with OCC or those without. **D** LRC stratified by the group of patients with OCC or those without

The median follow-up period was 40.5 months (range, 2–129). The 3-year OS, DFS, and LRC rates were 67.5%, 57.8%, and 82.7%, respectively (Fig. 2A). Comparing the oral cavity carcinoma (OCC) group and those without, the 3-year OS rates in the OCC and those without were 59.9% and 76.4%, respectively (*p* = 0.23; Fig. 2B). The 3-year DFS of the two groups were 57.3% and 58.2%, respectively (*p* = 0.78; Fig. 2C). The 3-year LRC of the two groups were 72.3% and 95.0%, respectively (*p* = 0.004; Fig. 2D). The 3-year LRC rate was significantly lower in the OCC group.

Owing to the statistically significant differences in LRC between the non-OCC and OCC groups, we compared patient backgrounds and treatment details between the two groups. The data are summarized in Tables 3 and 4. Among the patient characteristics, the clinical stage and T classification were more advanced in the non-OCC group, and the proportions of females and patients with ECEs were higher in the OCC group. We found no significant intergroup differences in the other factors such as age and positive margin. Among the treatment details, radiation dose showed no significant differences. For the radiation field, six patients in the OCC group received unilateral radiotherapy. No statistically significant difference in boost irradiation or concomitant chemotherapy was noted between the two groups, but the chemotherapy regimens were diverse and not uniform. CDDP tended to be used more frequently in the OCC group.

**Table 3:** Comparison of patient characteristics between non-OCC group and OCC group

	Non-OCC group (*n* = 40)	OCC group (*n* = 50)	*p* value
Primary site			
	Hypopharyngeal, 27 (68%)	Tongue, 42 (84%)	
	Oropharyngeal, 9 (22%)	Oral floor, 5 (10%)	
	Laryngeal, 4 (10%)	Gingival, 3 (6%)	
Age, years			0.93
Median (range)	68 (43–87)	65 (27–88)	
Sex			0.027
Male	37 (93%)	37 (74%)	
Female	3 (8%)	13 (26%)	
Stage			0.002
II	0 (0%)	1 (2%)	
III	0 (0%)	10 (20%)	
IVa	39 (98%)	39 (78%)	
IVb	1 (2%)	0 (0%)	
T classification			0.003
T1	1 (2%)	12 (24%)	
T2	8 (20%)	12 (24%)	
T3	8 (20%)	13 (26%)	
T4	23 (58%)	13 (26%)	
N classification			0.24
N0	2 (5%)	5 (10%)	
N1	3 (7%)	9 (18%)	
N2a	2 (5%)	0 (0%)	
N2b	21 (53%)	26 (52%)	
N2c	11 (27%)	10 (20%)	
N3	1 (3%)	0 (0%)	
Extracapsular extension			0.016
Yes	3 (7%)	14 (28%)	
No	37 (93%)	36 (72%)	
Positive surgical margin			> 0.999
Yes	4 (10%)	4 (8%)	
No	36 (90%)	46 (92%)	

*OCC* oral cavity carcinoma

**Table 4:** Comparison of treatment details between the non-OCC and OCC groups

	Non-OCC group (*n* = 40)	OCC group (*n* = 50)	*p* value
Radiation dose			0.152
Median (range)	50.4 (50.4–66.6)	50.4 (50.4–66.6)	
Radiation field			0.032
Bilateral	34 (85%)	31 (62%)	
Bilateral + boost	6 (15%)	13 (26%)	
Unilateral	0 (0%)	5 (10%)	
Unilateral + boost	0 (0%)	1 (2%)	
Boost irradiation			0.2
Yes	6 (15%)	14 (28%)	
No	34 (85%)	36 (72%)	
Concurrent systemic chemotherapy			> 0.999
Yes	30 (75%)	38 (76%)	
No	10 (25%)	12 (24%)	
Chemotherapy regimen			< 0.001
CDGP + 5FU	24 (80%)	8 (21%)	
CDGP + DTX	0 (0%)	6 (16%)	
CDDP	2 (7%)	11 (29%)	
CDDP + 5FU	0 (0%)	9 (24%)	
CDDP + DTX	0 (0%)	1 (3%)	
CBDCA	0 (0%)	2 (5%)	
DTX	4 (13%)	1 (3%)	

*OCC* oral cavity carcinoma, *CDGP* nedaplatin, *5FU* 5-fluorouracil, *DTX* docetaxel, *CDDP* cisplatin, *CBDCA* carboplatin

#### Patterns of recurrence

**Fig. 3:**
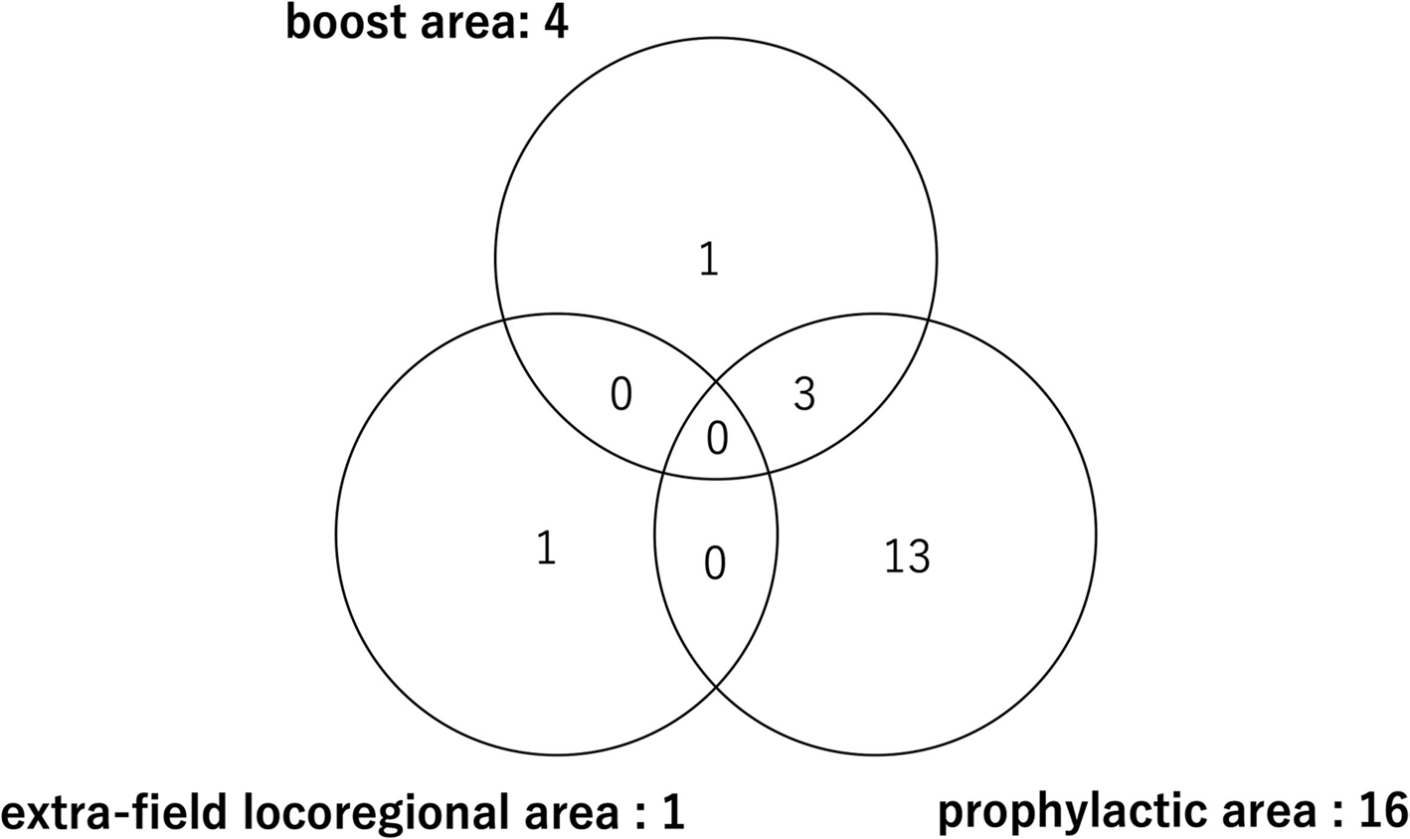
Venn diagram representing the pattern of locoregional failure

The patterns of the first relapse in the two groups are summarized in Table 5. In total, 43 patients experienced disease relapse after treatment, including 18 locoregional recurrences and 29 distant metastases. Statistically significant differences in locoregional and prophylactic area failures were noted between the two groups (*p* = 0.009 and *p* = 0.045). Figure 3 outlines the patterns of locoregional recurrence; four patients developed a boost area recurrence; 16, locoregional recurrence; and 1, extra field recurrence. Among the patients with a boost recurrence, three had a synchronous locoregional recurrence. Among the nine patients with oropharyngeal carcinoma, one patient who was p16-negative had locoregional recurrence whereas two patients with unknown p16 status had distant metastases. The only patient who was p16-positive did not have recurrence.

**Table 5:** Pattern of first failure

	Non-OCC (*n* = 40)	OCC (*n* = 50)	*p* value
	*n* (%)	*n* (%)	
Any failure	21 (53)	22 (44)	0.525
Locoregional failure	3 (7)	15 (30)	0.009
Boost area failure	0 (0)	4 (29)	0.27
Prophylactic area failure	3 (7)	13 (26)	0.045
Extra-field locoregional failure	0 (0)	1 (2)	> 0.999
Distant failure	18 (45)	11 (22)	0.025

*OCC* oral cavity carcinoma

The clinicopathological factors influencing the locoregional outcomes are listed in Table 6. Univariate analysis indicated that the tumor site influenced locoregional failure in the OCC group (hazard ratio = 5.29, 95% confidence interval = 1.41–19.8, *p* = 0.01). In the multivariate analysis, since 18 patients had a locoregional recurrence, two variables were used, namely, tumor site and the presence of concurrent chemotherapy, on the basis of previous studies and medical perspectives [7–10]. Furthermore, lower locoregional control was associated with oral cancer.

**Table 6:** Locoregional analysis

		Univariate analysis			Multivariate analysis		
		HR	95% CI	*p* value	HR	95% CI	*p* value
Age	< 65	1		0.34			
	≧ 65	1.33	0.46–3.82				
Sex	Male	1		0.58			
	Female	0.7	0.20–2.50				
Tumor site	Non-OCC	1		0.01	1		0.009
	OCC	5.29	1.41–19.8		5.24	1.51–18.2	
T stage	T1‑2	1		0.44			
	T3‑4	0.63	0.23–1.90				
N stage	N0‑1	1		0.58			
	N2a‑3	1.42	0.41–4.92				
Extracapsular extension	No	1		0.69			
	Yes	1.3	0.37–4.59				
Positive/closed margin	No	1		0.21			
	Yes	2.7	0.58–12.5				
Boost irradiation	No	1		0.53			
	Yes	1.46	0.45–4.75				
Concurrent chemotherapy	No	1		0.33	1		0.34
	Yes	0.57	0.19–1.76		0.62	0.23–1.66	

*OCC* oral cavity carcinoma, *HR* hazard ratio, *CI* confidence interval

#### Adverse events

Adverse events are summarized in Table 7. Acute adverse events of grade 3 or higher were mucositis, dermatitis, dysphagia, leukopenia, thrombocytopenia, and no others.

**Table 7:** Adverse events (*n* = 90)

	Acute	Late
	*n* (%)	*n* (%)
Dermatitis		
Grade 3 or higher	8 (9%)	0 (0)
Mucositis		
Grade 3 or higher	22 (24%)	0 (0)
Dysphagia		
Grade 3 or higher	10 (11%)	3 (3%)
Leukopenia		
Grade 3 or higher	27 (30%)	0 (0)
Thrombocytopenia		
Grade 3 or higher	4 (4%)	0 (0)

The only severe late adverse event was dysphagia and no others. Three patients were dependent on a PEG tube 12 months after the treatment. There were no cases of severe toxicities associated with flap reconstruction.

## Discussion

In this study, we analyzed the outcomes of postoperative radiotherapy performed at our institution. Although the irradiation dose used at our institution was relatively lower than that employed in Western countries, the treatment outcomes appeared comparable to previous findings [1, 2, 7, 9, 11, 12] (Table 8). Alternatively, locoregional control was significantly worse in the patients with than in those without OCC, even though we delivered the same radiation dose in both groups. In the current global standard for postoperative radiotherapy for head and neck cancers, the treatment intensity does not differ between OCC and non-OCC. However, several previous studies reported poor locoregional control of OCC in head and neck cancers, which suggests the limitations of the current treatments [9, 11, 13]. Considering the poor locoregional control observed in patients with OCC in this study, higher treatment intensity may be required for this malignancy, such as dose escalation in the cervical prophylactic area.

**Table 8:** Outcome of postoperative chemoradiotherapy in HNSCC patients by previous reports

Author	Year	No. of patients	Risk	ECE (%)	Treatment	RT (Gy)	LRC or LRR (%)	OS (%)
			Close/positive margin (%)				(year)	(year)
Bernier et al.	2004	167	31	61	CRT	66	LRR 18 (5)	53 (5)
		167	26	53	RT	66	LRR 31 (5)	40 (5)
Cooper et al.	2004	206	17	< 83	RT	60‑66	82 (2)	56 (3)
		210	19	< 81	CRT	60‑66	72 (2)	47 (3)
Yao et al.	2005	49	NR	NR	CRT	54‑66	82 (2)	NR
Chan et al.	2013	180	17	34	CRT	54‑66	78 (2)	65 (2)
Ooishi et al.	2016	122	30	77	RT/CRT	66	52 (3)	59 (3)
Makita et al.	2017	89	37	81	RT/CRT	60 (median)	83 (2)	72 (2)
Present	2021	90	9	19	RT/CRT	50.4 (median)	82.7 (3)	67.5 (3)

*HNSCC* head and neck squamous cell carcinoma, *ECE* extracapsular extension, *RT* radiation therapy, *CRT* chemoradiotherapy, *LRC* locoregional failure, *LRR* locoregional recurrence, *OS* overall survival, *NR* not reported

The present study included only the patients who received 3D-CRT, and those who received IMRT were excluded due to the very small number of IMRT cases. IMRT is currently accepted for patients with head and neck cancer because it allows increasing the dose while reducing the risk of adverse events. However, there are several challenges associated with the administration of IMRT, especially in postoperative situations. First, the tumor has been surgically removed; therefore, the CTV for IMRT is often challenging to determine. Second, IMRT may increase the risk of marginal recurrence by excessively narrowing the target volume [9, 11, 13]. Although these challenges remain, with IMRT, the dose can be escalated in the cervical prophylactic area without increasing the dose to the surrounding normal tissue such as the mandible or salivary glands. It appears likely that dose escalation in the prophylactic area in patients of OCC will improve the LRC rate. Further research is needed to determine whether this strategy will improve the LRC rate.

Acute and late adverse events were also comparable to or less severe than those identified in previous studies [9–12, 14]. In head and neck cancer, in particular, long-term PEG tube dependence is often a problem [15, 16], but few patients required a PEG tube for longer than 1 year in our study.

This study has several limitations. First, in this study, the proportion of patients with close/positive margins or ECEs was smaller than those in previous studies, which might have led to overestimation of the treatment results. Indeed, the number of high-risk cases was small, but of the 40 patients in the non-OCC group, only three (8%) had a locoregional recurrence, all in the prophylactic area. No recurrence was observed in the boosted areas. Therefore, especially for the non-OCC group, we believe that it might be possible to reduce the irradiation dose of the prophylactic area. Second, this study was a single-institution retrospective experience, and the small sample size might be insufficient for drawing definitive conclusions. Third, adverse events could not be thoroughly assessed, and they were likely underestimated because of the study’s retrospective nature. Fourth, because the otolaryngology or oral surgery department handled chemotherapy, there was no unified systemic chemotherapy regimen. The chemotherapy regimen was not standardized, which might have affected the results. Prospective clinical trials will be needed to overcome these limitations.

## Conclusions

This study suggests that de-escalation of the radiation dose in patients with non-OCC can potentially reduce the severe adverse events of irradiation while ensuring its effectiveness. However, in patients with OCC, it might be necessary to increase the radiation dose.

## Data Availability

The datasets used and analyzed during the current study are available from the corresponding author on reasonable request.

## Abbreviations

HNSCC: Head and neck squamous cell carcinoma
EORTC: European Organization for Research and Treatment of Cancer
RTOG: Radiation Therapy Oncology Group
ECE: Extracapsular extension
NCCN: National Comprehensive Cancer Network
PEG: Percutaneous endoscopic gastrostomy
IMRT: Intensity-modulated radiation therapy
CT: Computed tomography
3DCRT: Three-dimensional conformal radiotherapy
CTV: Clinical target volume
OS: Overall survival
DFS: Disease-free survival
LRC: Locoregional control
OCC: Oral cavity carcinoma
